# The Role of Semi‐Wild Habitats in the Physical Conditions of Juvenile Alligators: Implications for Conservation

**DOI:** 10.1002/ece3.72451

**Published:** 2025-11-17

**Authors:** Lulu Cui, Qin Wang, SiQing Sun, WenGang Li, Lingyi Li, Ke Sun, Yongkang Zhou, Genjun Tu, RuoYa Liu, Lei Li, Zhenpeng Yu, Chongzhi Zhang, Xiaobing Wu, Tao Pan

**Affiliations:** ^1^ College of Life Sciences Anhui Normal University Wuhu Anhui China; ^2^ The Anhui Provincial Key Laboratory of Biodiversity Conservation and Ecological Security in the Yangtze River Basin Wuhu Anhui China; ^3^ School of Health Science and Engineering Ma'anshan University Ma'anshan Anhui China; ^4^ National Long‐Term Scientific Research Base for Chinese Alligator Artificial Breeding and Protection in Anhui Anhui Research Center for Chinese Alligator Reproduction Xuancheng Anhui China

**Keywords:** juvenile alligators, microbiome, physical condition, semi‐wild

## Abstract

The number and survival rate of juveniles play a key role in the recovery of Chinese alligators ( *Alligator sinensis* ). The differences between artificial and semi‐wild environments can directly affect the growth and development of juvenile alligators. This study analyzed the physical conditions (lengths and weights) and cloacal microbial communities of juvenile alligators in both artificial breeding (DJ, ZX) and semi‐wild (GJM) environments to reveal the significant effects of environmental pressure on their physiological state and microbiome. The results revealed that the body length (23.15 ± 1.06 cm) and weight (22.8 ± 3.08 g) of juvenile alligators in the GJM were significantly lower than those in the artificial environment (body length = 29.5 cm, weight = 68.6 g; *p* < 0.01). Moreover, the microbial α diversity (ACE, Shannon) of the GJM was significantly reduced, and the community structure was significantly separated (NMDS analysis), suggesting that the pressure of the semi‐wild environment inhibited growth. In terms of microbial composition, the relative abundance of Actinobacteria in the GJM group was significantly, increased, whereas that of Bacteroidetes was decreased, and Microbacteria and Cyanobacteria were unique; at the genus level, environmentally specific marker genera were identified (such as *Limnohabitans* and *Pseudomonas* in GJM and *Fluviicola* and *Deinococcus* in the artificial environments). LEfSe analysis further elucidated the differential marker microbiota (such as Actinobacteria/Cyanobacteria in GJM). In summary, stress (such as food shortages) in semi‐wild environments affects the growth and development of juvenile alligators by changing their microbial communities (such as enriched actinomycetes), This finding provides a microbial ecological basis for optimizing the conservation strategy of the Chinese alligator.

## Graphical abstract

It plays a key role in the recovery of the Chinese alligator population with respect to the number and survival rate of juveniles. The differences between artificial and semi‐wild environments can directly affect the growth and development of juvenile alligators. This study analyzed the physical condition (length and weight) indicators and cloacal microbial communities of juvenile alligators in artificial breeding (DJ, ZX) and semi‐wild (GJM) environments to reveal the significant effects of environmental pressure on their physiological state and microbiome. The results showed that the body length (23.15 ± 1.06 cm) and weight (22.8 ± 3.08 g) of juvenile alligators in the GJM were significantly lower than those in the artificial environment (body length = 29.5 cm, weight = 68.6 g; *p* < 0.01). At the same time, the microbial α diversity (ACE, Shannon) of the GJM was significantly reduced, and the community structure was significantly separated (NMDS analysis), suggesting that the pressure of the semi‐wild environment inhibited growth. In terms of microbial composition, the relative abundance of Actinobacteria in the GJM group increased significantly, while that of Bacteroidetes decreased, and Microbacteria and Cyanobacteria were unique; at the genus level, environmental‐specific marker genera were identified (such as Limnohabitans and Pseudomonas in GJM and Fluviicola and Deinococcus in artificial environments). LEfSe analysis further clarified the differential marker microbiota (such as Actinobacteria/Cyanobacteria in GJM). In summary, stress (such as food shortage) in semi‐wild environments affects the growth and development of juvenile alligators by changing their microbial communities (such as enriched actinomycetes), providing a microbial ecological basis for optimizing the conservation strategy of the Chinese alligator.

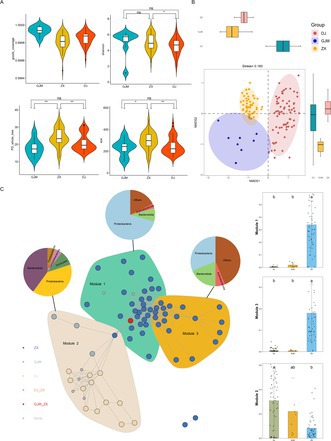

## 1 Introduction

### 1.1 Health of Juvenile Alligator and Chinese Alligator Population Recovery

The Chinese alligator ( *Alligator sinensis* ) is an ancient and endangered species of freshwater crocodile that is unique to China. In recent years, threats such as habitat loss, environmental pollution and hunting (Wan et al. 2013) have severely damaged the habitat of the wild population of this species. Consequently, its distribution range has shrunk dramatically, and by the late 1980s and early 1990s, the Chinese alligator had reached a dotted distribution. Wild population monitoring data have shown that the number of wild Chinese alligators currently remains extremely low (Pan et al. 2019). The key to the recovery of the Chinese alligator population lies in increasing the number and survival rate of the young alligators, as the health status of young alligators directly affects individual growth and development and population rejuvenation. It is, therefore, considered crucial to the protection of germplasm resources. Captive breeding is an important strategy for the protection of endangered species, that has played a key role in the protection of the Chinese alligator (Gray 1985). In this study, in order to help the artificially bred young crocodiles adapt quickly to the wild environment after release, a semi‐wild environment (GJM) was set up outside the artificial breeding environment (ZX and DJ), and a pilot project on the wild breeding of young crocodiles was conducted.

### 1.2 Microbiota–Host–Environment Relationships

The intestine is the core organ of digestion and immune defense and is colonized by a huge microbial flora. In reptiles, the intestinal microbiota participates in the endocrine regulation of nutrient digestion and absorption, exerting a significant impact on the growth, development, immunity, and metabolism of the host (Eckburg et al. 2005; Hanning and Diaz‐Sanchez 2015). Research suggests that microorganisms can promote the growth of young alligators (Li et al. 2025). The intestinal microbiota is a core factor affecting health and disease, and its composition and function are shaped jointly by the host's genetic and environmental factors (Hanning and Diaz‐Sanchez 2015). Healthy intestinal flora can help the host cope with changes in the external environment, and also play an important role in preventing the development of diseases, such as neurodevelopmental disorders and cancer (Lee et al. 2021; Siddiqui et al. 2021). However, dysbiosis is closely related to various systemic diseases and can lead to changes in host immunity, nutrition, and even behavior (Nie et al. 2019). The existing research on intestinal microbes includes many vertebrate groups, such as mammals, birds, fish, amphibians, and reptiles (Thaiss et al. 2016; Hird et al. 2015). The cloacal, tract is the common opening of the digestive, urinary, and reproductive systems of reptiles, which is located at the end of the rectum, and its microbial community appropriately reflects the state of the end of the intestine. The cloaca also exhibits a greater frequency of exchanging substances with the outside world and can, therefore, reflect the relationship between the host and the environment better than the intestines. Cloaca sampling is therefore, a common method for studying rare species, and its non‐invasive nature can minimize animal stress and injury risks.

The health of young alligators is generally considered one of the key targets in the rejuvenation of the Chinese alligator population. The microbiome of young alligators can help the animals in their healthy growth. The environment plays a significant role in shaping host microorganisms. In this context, the present study used one‐year‐old alligators from the Anhui Research Center for Chinese Alligator Reproduction (ZX), Gaojingmiao area (GJM), and Wuhu Dajiang Breeding Base (DJ) as research subjects, and systematically compared the effects of different environments on microbial communities and body size characteristics, revealing the shaping effect of the geographical environment on microorganisms and the intrinsic relationship between physical conditions and microorganisms. The findings of this study would provide a scientific basis for optimizing the breeding environment of alligators.

## 2 Materials and Methods

### 2.1 Rearing Environment and Sample Collection

In order to improve the population of Chinese alligators and their ability to adapt to the wild, this study focused primarily on the artificial breeding areas (DJ and ZX, located in Wuhu City and Xuancheng City, respectively) and semi‐wild breeding areas (GJM, located in a wild protected region) in China. The artificial breeding areas have a complete breeding system, which includes a temperature and humidity control system, artificial disinfection (breeding pond with 0.3% potassium permanganate aqueous solution) three times a week, artificial feeding mainly with feed provided twice a day, and a breeding pool with a completely hardened tile floor, resulting in extremely low environmental pressure. The semi‐wild breeding area has less human intervention, with the use of natural temperature, humidity, and light, and no artificial disinfection measures. Only live foods such as frogs, fish, and snails are supplemented twice a month. Thus, environmental pressure is relatively high. On the same day, the researchers measured the individual physical signs (body lengths and weights) in three areas (DJ: *n* = 10; ZX: *n* = 14; GJM: *n* = 8) and collected all cloacal microbial samples (DJ: *n* = 52; ZX: *n* = 56; GJM: *n* = 12) for subsequent analysis.

### 2.2 DNA Extraction and Sequencing

Total microbial genomic DNA was extracted from the sampling swabs according to the instructions provided with the FastDNA Soil Centrifugation Kit (MP Biomedicals, Solon, GA, USA). DNA integrity and purity were monitored on 1% agarose gels. The DNA concentration and purity were determined with a Qubit 3.0 (ThermoFisher Scientific, Waltham, USA) and NanoDrop One (ThermoFisher Scientific, Waltham, USA), and the DNA samples were then stored at −80°C. In accordance with the methods described in previous studies, the 16S rRNA gene sequences were PCR amplified by targeting the V3–V4 region using a set of forward and reverse primers: Pro_341F (5′‐ATCCTACGGGAGGCAGCA‐3′) and Pro_806R (5′‐GGACATACHVGGGTWTCTAAT‐3′) (Takai and Horikoshi 2000). The amplified DNA was submitted to Magigene Biotechnology Co. Ltd., Guangzhou, China, for high‐throughput sequencing. The 16S amplicon sequencing was completed using the Illumina MiSeq sequencing platform (Illumina, San Diego, California, USA).

### 2.3 16S Data Sequencing Analysis

The raw data from 16S sequencing were quality‐controlled and filtered using fastp software to obtain the clean data. DADA2 was used to merge the clean data, following which the chimeras and primers were removed, and the obtained sequences were clustered to generate ASVs. Qiime2 was used to align the ASVs to the Silva database to generate a classification table. Finally, chloroplast and mitochondrial contamination was removed, and the rare ASVs (0.00005) of all tags, single or relatively low‐abundance sequences usually representing the erroneous sequences (such as chimeras), were finally acceptable to artificially remove from the data set (Cao et al. 2021; Auer et al. 2017); however, there is no universally accepted threshold, and 0.00005 (0.005%) is usually recommended in previous studies (Bokulich et al. 2013). Accordingly, the data were filtered out to generate a clean bacterial classification table for subsequent analysis.

### 2.4 Statistical Analysis of Data

The body length and weight data were analyzed using a t‐test for significant differences, and a linear fitting analysis was performed. Based on the ASV cluster table, QIIME2 was used to calculate the α (such as the Shannon index) and β diversity (Bray–Curtis distance), and box plots and non‐metric multidimensional scaling (NMDS) scatter plots were generated for an intuitive display of the differences in community structure. The relative abundance of microorganisms at the phylum/genus level was statistically analyzed by group, and the top 10 dominant groups in each group were screened. LEfSe (LDA score ≥ 3) was performed to identify the species based on the significant differences between groups; the indicspecies R package was used for the macronetwork analysis to reveal the symbiotic pattern of the species and environmental factors. Macronetwork analysis links differential analysis with network analysis, performs network analysis on all encapsulated samples, and highlights the differential species, thereby allowing the exploration of the network modules that respond to different treatments.

## 3 Results

**FIGURE 1:**
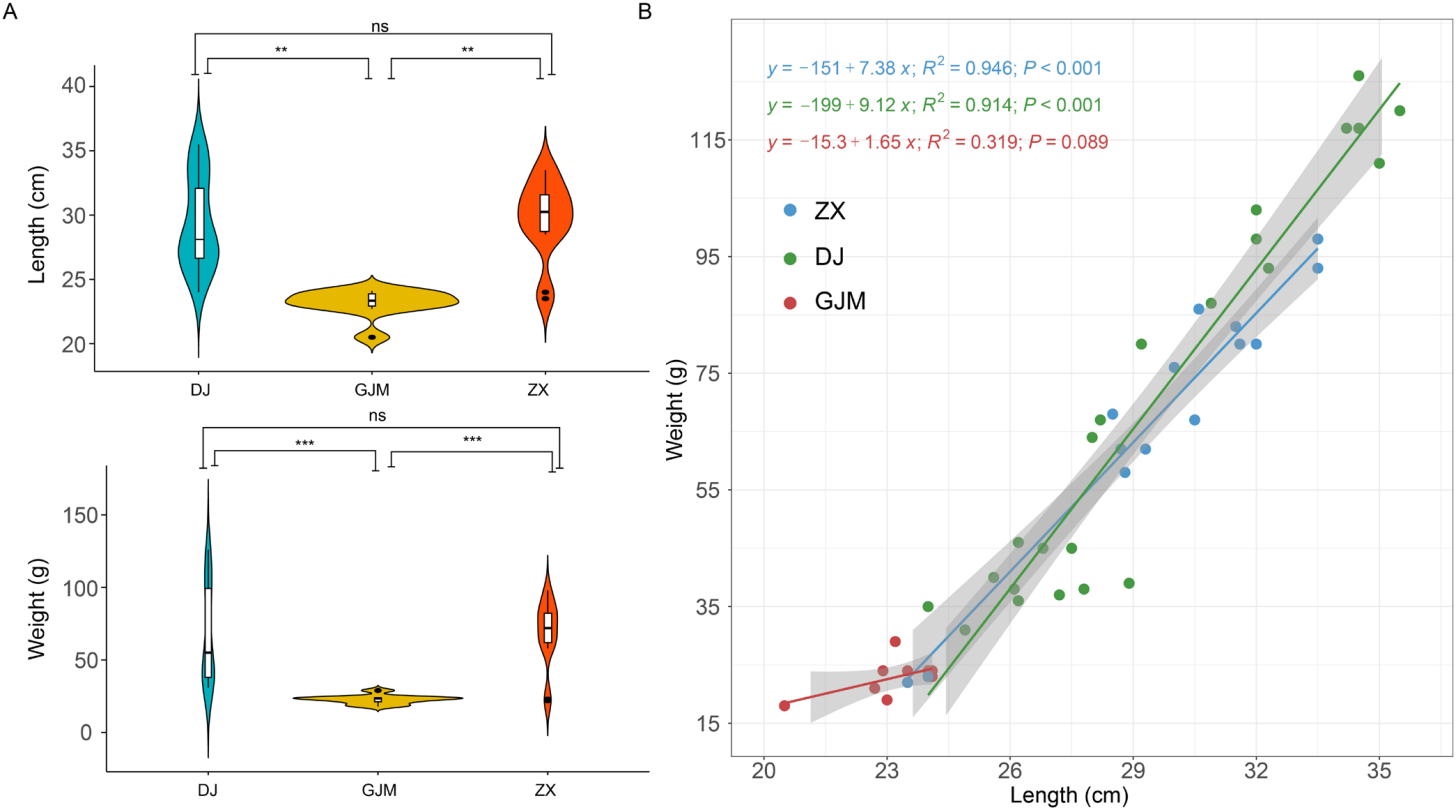
Body size differences and body size parameter fitting of the individuals in different rearing environments. (A) Analysis the of body size parameter differences among young alligators in different rearing environments, with the horizontal axis representing the grouping and the vertical axis representing the body condition parameters (body length and weight). The white dots denote the mean, the outer contour indicates the sample distribution, the upper side of the middle black box represents the upper 1/4, and the lower side represents the lower 1/4. An independent sample *t*‐test was performed between groups, where “***” means *p* < 0.001, “**” means *p* < 0.01, and “ns” means *p* > 0.05. (B) Linear fitting was performed for the body length and weight data of individuals in different environments, and the linear relationship function and R2 were calculated.

In order to explore the effects of different growth environments on the growth of young alligators, the differences in the body lengths and weights were first compared among the young alligators in different environments. The results revealed that the body lengths (*n* = 10, mean ± SD = 23.15 cm ±1.06) and weights (*n* = 10, mean ± SD = 22.8 g ±3.08) of the young alligator in the GJM environment were significantly (*p* < 0.01) lower than those in the DJ environment (*n* = 10; body lengths: 29.36 cm ±3.55; weights: 68.75 g ±33.85) and the ZX environment (*n* = 14; body lengths: 29.71 cm ±3.00; weights: 68.43 g ±22.77) (Figure 1A). However, there were no significant differences in body length or weight between the DJ and ZX environments. Linear correlation analysis of the body length and weight of the individuals in the three environments revealed a strongly significant correlation (*p* < 0.001) between the body length and weight of individuals in ZX (*R* ^2^ = 0.946 [6.28, 8.49]) and DJ (*R* ^2^ = 0.914 [7.88, 10.36]), whereas there was no significant correlation (*p* > 0.05) in GJM (*R* ^2^ = 0.319 [−031, 3.1]) individuals (Figure 1B). These results indicated that the GJM environment is not conducive to the normal growth of young crocodiles compared to the DJ and ZX environments.

**FIGURE 2:**
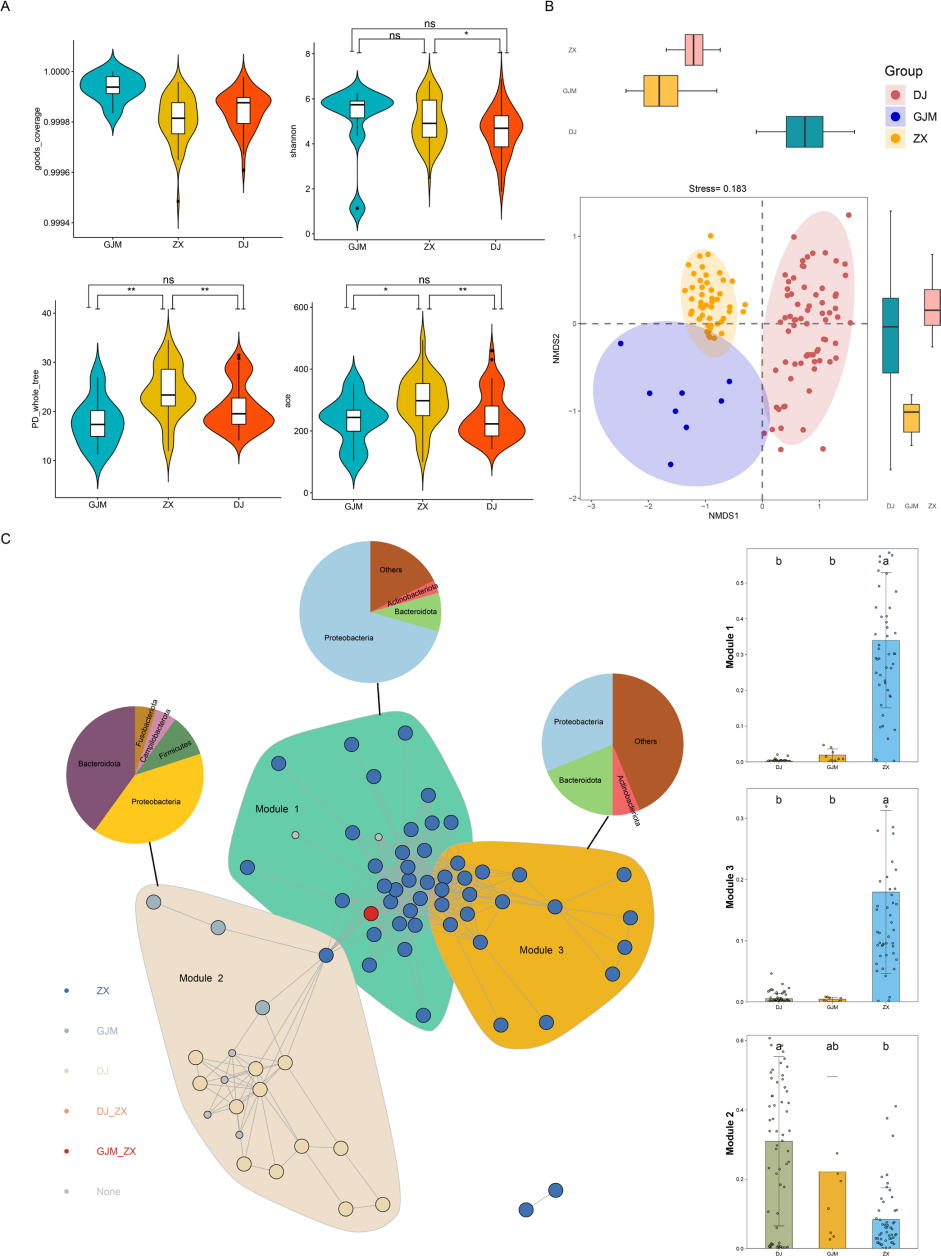
Differences in the microbial diversity in the cloaca of young alligators in different environments. (A) Comparison of microbial α diversity between the groups in different environments, with the horizontal axis representing the group and the vertical axis representing the α index (observation norm, Chao1, Shannon, and Simpson). The white dots denote the means, the outer contour indicates the sample distribution, the upper side of the middle black box represents the upper 1/4, and the lower side represents the lower 1/4. Independent sample t tests were performed between groups, where “**” indicates *p* < 0.01, “*” indicates 0.01 < *p* < 0.05, and “ns” indicates *p* > 0.05. (B) Non‐metric multidimensional scaling analysis based on the ASV table using the Bray–Curtis dissimilarity distance revealed that the samples could be significantly clustered into three categories according to different environments. Macro network analysis of the age gradient. (C) Networks of different ages are marked with different colors, different modules are marked using different colors, and pie charts are used to show the phylum‐level compositions of different modules.

In order to analyze the differences in microbial community structure in different environments, the α diversity indices (including the ACE, Shannon, PD whole tree, and Good's Cover indices) were calculated based on relative abundance. The results revealed that the Good's Cover values of all the samples were greater than 0.998, indicating that the sequencing depth met the analysis requirements. The ACE and PD whole‐tree indices of the GJM environment were significantly lower than those of the ZX, and there was no significant difference in the Shannon index between ZX and GJM. The results revealed that the microbial richness of the GJM samples was significantly lower than that of the ZX samples, but there was no significant difference in uniformity (Figure 2A). Non‐metric multidimensional scaling (NMDS) analysis based on the Bray–Curtis distance (stress = 0.12) revealed that individuals in different rearing environments formed significantly separated clusters in the two‐dimensional sorting space, indicating that their microbial community compositions differed significantly (Figure 2B). The symbiotic network constructed through the metagenomic network analysis (co‐occurrence *r* > 0.6, *p* < 0.01) revealed that microbial interactions formed three functional modules, among which Bacteroidota, Proteobacteria, and Firmicutes presented extensive topological connections between modules, suggesting that these groups played a core role in the ecological function of the community (Figure 2C).

**FIGURE 3:**
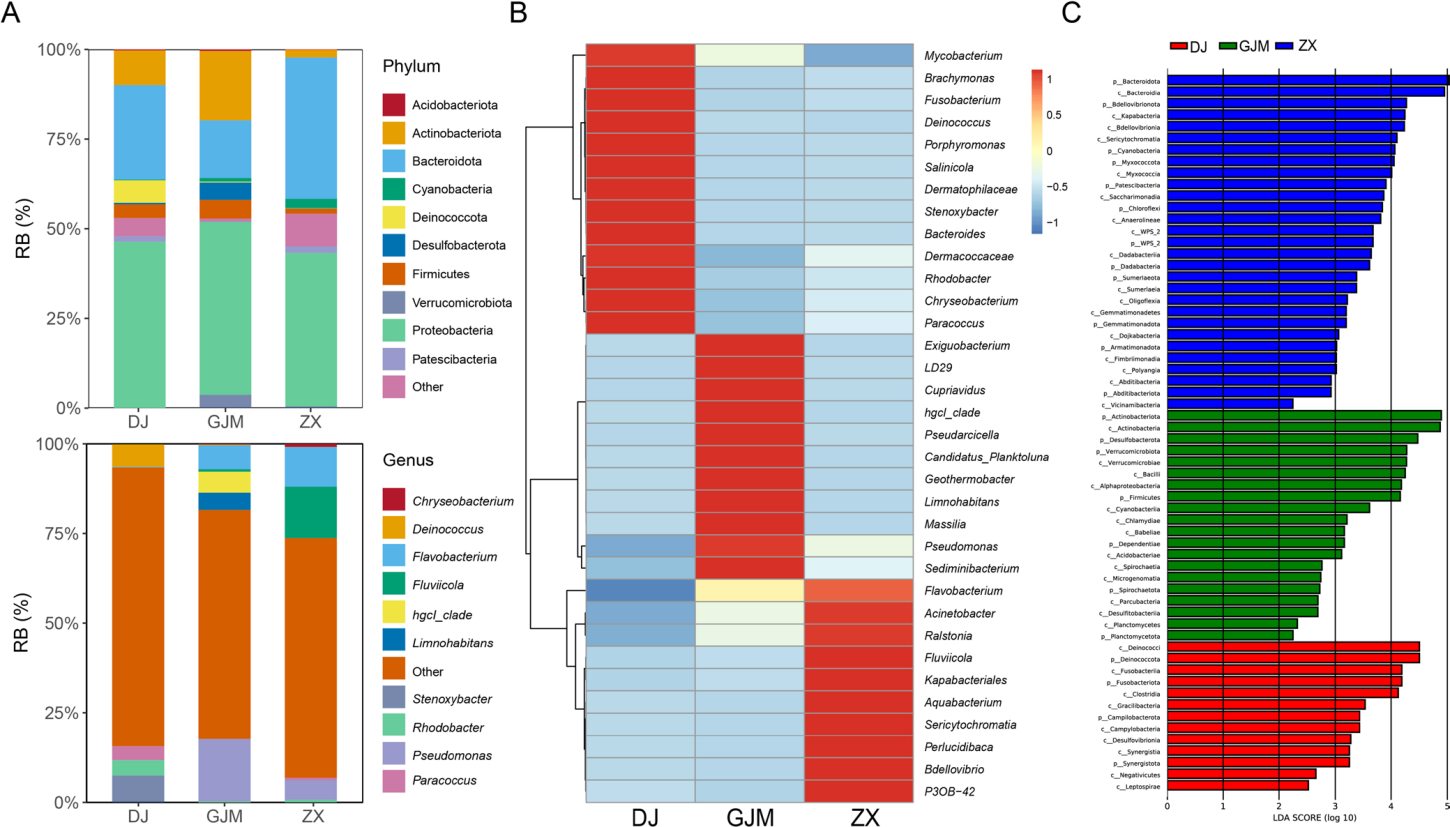
Analysis of the host microbial composition. (A) Statistics of the top 10 microbial phyla and genera in different groups. The horizontal axis represents the grouping; and the vertical axis represents the relative abundance of microorganisms. Each color represents the phylum and genus levels of the microorganism. (B) The top 34 genera of samples in the three environments are displayed in the form of a heatmap. (C) Key differential taxonomic units of microorganisms in different groups. LEfSe was used to compare the differences in the microorganisms in the different groups. The bar graph illustrates the bacteria with significant differences in their relative abundance. The other bar graph represents the linear discriminant analysis (LDA) score.

In view of the differences in the microbial composition under different feeding environments, statistical analysis of the different groups was performed at the phylum and genus levels. At the phylum level, the phylum with the highest relative abundance in all three groups was Proteobacteria, followed by Actinobacteria and Bacteroidetes. There was no significant difference in the relative abundance of Proteobacteria among the three groups. The relative abundance of Bacteroidota in the GJM group was significantly lower than that in the DJ and ZX groups, whereas the relative abundance of Actinobacteria was significantly greater than that in the DJ and ZX groups. In addition, Patescibacteria and Cyanobacteria were detected only in the GJM group. At the genus level, the microbial composition varied greatly among the groups; while *Pseudomonas* was present in all three groups, its relative abundance in the GJM group was significantly greater than that in the other two groups. *Limnohabitans* and *hgcI_clade* were present only in the GJM. *Fluviicola* had the highest abundance in ZX. *Deinococcus* had the highest abundance in DJ. These results showed that the microbial composition at the genus level in different environments differs significantly, and this difference can reflect the suitability of the environment to a certain extent (Figure 3A).

In order to further analyze the differences in the microorganisms between different environments, cluster analysis was performed using the top 35 genera in terms of relative abundance. The genera could be significantly clustered into three categories corresponding to the different environments. The high‐abundance genera (relative abundance > 2%) in the DJ (artificial environment) group included *Brachymonas* (3.44%), *Fusobacterium* (3.12%), *Deinococcus* (6.32%), *Porphyromonas* (2.82%), *Dermatophilaceae* (2.91%), *Stenoxybacter* (7.51%), *Bacteroides* (3.28%), *Rhodobacter* (4.19%), *Chryseobacterium* (4.28%), *Paracoccus* (3.89%). The high‐abundance genera of GJM included *Exiguobacterium* (4.25%), *LD29* (2.49%), *hgcI_clade* (5.84%), *Pseudarcicella* (2.13%), *Candidatus_Planktoluna* (3.81%), *Geothermobacter* (3.71%), *Limnohabitans* (4.80%), *Massilia* (2.84%), *Pseudomonas* (17.37%), and *Sediminibacterium* (3.23%). The high‐abundance genera of ZX (artificial environment) included *Flavobacterium* (6.81%), *Acinetobacter* (2.69%), *Fluviicola* (14.27%), *Kapabacteriales* (3.62%), *Aquabacterium* (4.31%), *Sericytochromatia* (2.56%), and *Bdellovibrio* (3.07%) (Figure 3B). In order to identify the key markers of the differences between the groups, the LDA discriminant analysis (LDA > 4) was performed. The results revealed that the DJ group had nine markers, including four phyla (Deinococci, Fusobacteriota, Campilobacterota and Synergistota) and five classes (Deinococci, Fusobacteriia, Clostridia, Gracilibacteria, and Campylobacterota). The GJM group had eight markers, including three phyla (Actinobacteriota, Desulfobacterota, and Verrucomicrobiota) and five classes (Actinobacteria, Verrucomicrobiota, Bacilli, Alphaproteobacteria, and Cyanobacteriia). ZX had five markers, including two phyla (Bacteroidota and Bdellovibrionota) and three classes (Bacteroidia, Kapabacteria, and Bdellovibrionia) (Figure 3C). According to these results, the key differential taxonomic units in different environments could be identified.

## 4 Discussion

This study compared the physical condition characteristics and the cloacal microbial community differences in young alligators bred in artificial breeding (DJ, ZX) and semi‐wild (GJM) environments. The goal was to analyze the impact of the environment on individual growth. Compared to a semi‐wild environment, artificial breeding conditions exerted a positive effect on the growth and physical condition of Chinese alligators, and the composition and structure of individual cloacal microorganisms differed significantly. However, the discrepancy between the body size data and the microbial data in this study could have affected the relationship between body size and environmental microbes to some extent. Therefore, to address this issue, findings from our previous research (Li et al. 2025) were introduced in the introduction section to demonstrate the correlation between body size and microbes, supporting this research from another perspective.

Many studies have shown that human activities significantly affect the migration, growth, and development, and enhance the reproduction of reptiles (Tan et al. 2023; Todd and Andrews 2008). Habitat loss caused by land use is considered to be the main reason for the decline in reptile populations (Amburgey et al. 2021). Changes at the individual level are highly valuable in the study of species population dynamics. On one hand, these changes reflect an individual's ability to adapt to the environment (Doherty et al. 2022; Pirotta et al. 2018). On the other hand, such changes can be used as indicators of habitat quality. At present, long‐term studies have systematically evaluated relationships among the physical condition decline, population changes and habitat conditions (Gardner et al. 2016; Pigeon et al. 2017). The results of this study revealed that the body size of young crocodiles in artificial environments was significantly larger than that in semi‐wild environments, indicating that artificial intervention is more conducive to the growth of young crocodiles. This may be because the constraints of various closely related stress factors in the semi‐wild environment (GJM), such as food scarcity, threats from natural enemies, and a lack of artificial shelter, decelerate the growth of young crocodiles, causing their body lengths and weights to be significantly lower than those in artificial breeding environments (DJ and ZX). In addition, and the length‐weight correlation is lost, which directly confirms the negative impact of wild pressure on growth.

Intestinal (and cloacal) microbes serve as the “second genome” of the host, and their composition and function are directly related to nutrient absorption, basal metabolism, and immune health of the host. The structure and composition of host microbes are directly affected by the living environment of the population. Xie et al. reported that captive breeding and artificial breeding of red‐crowned cranes ( *Grus japonensis* ) significantly altered the structure of the intestinal microbiota (Xie et al. 2016). The intestinal microbial composition of crowned lizards ( *Anolis cristatellus* ) living in cities was reportedly similar to that of humans and contained bacterial strains associated with human urbanization (Dillard et al. 2022). The beta diversity of the intestinal microbes of Sceloporus lizards in different regions has been reported to be significantly different (Bunker and Weiss 2022). Although the abundances of major bacterial phyla and pathogenic genera in the feathers and nests of urban and rural birds are similar, the bacterial community richness and potential pathogen load of birds in urban habitats are relatively high, which may be one of the reasons for the difference in bacterial communities between the two locations. Moreover, the similarity of bacterial communities between nests and their residents suggests the transmission of bacteria between them (Stephens et al. 2021). The results of this study also showed that individuals in different environments have different microbial compositions. In the GJM region, which has greater environmental pressure, the relative abundance of Actinobacteria was significantly greater than that in the DJ and ZX regions, and Patescibacteria and Cyanobacteria were detected only in the GJM region. Studies have also reported that the decreased Firmicutes/Bacteroidetes ratio in hibernating butterfly lizards in a state of long‐term fasting helps them live through the winter months, as the bacteria of the phylum Bacteroidetes use the host‐derived mucin glycans in the absence of dietary substrates (Zhu et al. 2024). These findings suggest that Actinobacteria may serve as relevant species indicative of the environmental stress caused by food scarcity, whereas Patescibacteria and Cyanobacteria may be relevant species indicative of the environment in the GJM region.

In summary, the differences in the body size of young alligators in the GJM region indicated that food‐deficient environments are not conducive to individual growth. Under the influence of environmental selection pressure, the young alligators in the GJM region exhibited a unique microbial community structure, with a relatively high abundance of Actinobacteria under food‐deficient environmental pressure, whereas Microbacteria and Cyanobacteria were detected only in the young alligators in this region and could be used as relevant environmental species in the GJM region. The significant differences in the microbial abundance across the GJM region suggested that food scarcity is a key factor affecting the growth of young alligators in semi‐wild environments. This also provides insights for improving the reintroduction efforts, focusing on further improving the food chain and web in the wild to ensure that the released alligators have access to plentiful food sources, helping them adapt more quickly to the wild.

## Author Contributions

**Lulu Cui:** investigation (equal), writing – original draft (equal). **Qin Wang:** data curation (equal), writing – original draft (equal), writing – review and editing (equal). **SiQing Sun:** investigation (equal), writing – original draft (equal). **WenGang Li:** investigation (equal). **Lingyi Li:** investigation (equal). **Ke Sun:** data curation (equal). **Yongkang Zhou:** investigation (equal). **Genjun Tu:** investigation (equal). **RuoYa Liu:** investigation (equal). **Lei Li:** investigation (equal). **Zhenpeng Yu:** investigation (equal). **Chongzhi Zhang:** investigation (equal). **Xiaobing Wu:** writing – review and editing (equal). **Tao Pan:** writing – review and editing (equal).

## Disclosure

*Publisher's Note*: All claims expressed in this article are solely those of the authors and do not necessarily represent those of their affiliated organizations, or those of the publisher, the editors, or the reviewers. Any product that may be evaluated in this article, or claim that may be made by its manufacturer, is not guaranteed or endorsed by the publisher.

## Ethics Statement

All experiments were approved by the University of Anhui Normal University Academic Ethics Committee (AHNU–ET2023003).

## Conflicts of Interest

The authors declare no conflicts of interest.

## Data Availability

All sequencing data generated in this study are available on CRA029582 (https://ngdc.cncb.ac.cn/gsa/search?searchTerm=CRA029582).
